# Breast cancer metastasizing to the contralateral axilla several years after treatment: A case report with literature review

**DOI:** 10.1016/j.ijscr.2021.105900

**Published:** 2021-04-27

**Authors:** Abdulwahid M. Salih, Zuhair D. Hammood, Marwan N. Hassan, Hiwa O. Baba, Aso S. Muhialdeen, Ismael Y. Abdullah, Berwn A. Abdulla, Fahmi H. Kakamad, Shevan M. Mustafa, Shvan H. Mohammed, Mohammed Q. Mustafa

**Affiliations:** a Faculty of Medical Sciences, School of Medicine, University of Sulaimani, Sulaimani, Kurdistan, Iraq; b Smart Health Tower, Madam Mitterrand Str, Sulaimani, Kurdistan, Iraq; c Kscien Organization, Hamdi Str, Azadi Mall, Sulaimani, Kurdistan, Iraq; d Faculty of Medical Sciences, School of Medicine, Department Cardiothoracic and Vascular Surgery, University of Sulaimani, Sulaimani, Kurdistan, Iraq; e Department of Medical Analysis, Tishk International University, Erbil, Kurdistan Region, Iraq

**Keywords:** Breast cancer, Contralateral spread, Ipsilateral spread

## Abstract

**Introduction and importance:**

Lymph node metastasis is the most prominent prognostic factor in breast cancer. The aim of this paper is to report a case of contralateral axillary lymph node metastasis (CAM) which look like metachronous initially, but histopathologicaly confirmed as synchronous CAM.

**Case presentation:**

A-44-year old female was a known case of left breast cancer five years prior to this presentation (T2,N2,M0, grade III, Triple negative, multifocal invasive ductal carcinoma). On follow up, multiple contralateral axillary suspicious lymph nodes were discovered. Fine Needle Aspiration Cytology from the lesion revealed grade III, Triple negative, invasive ductal carcinoma consistent with metastasis from the left invasive ductal carcinoma. Bilateral mastectomy and right axillary dissection were performed. The histopathological examination and immunohistochemistry showed left breast recurrent 0.5 cm grade III, Triple negative invasive ductal carcinoma.

**Discussion:**

If a cancer is found in the contralateral axilla, three main potential sources should be considered: contralateral spread from the original breast tumor, metastasis from an occult primary in the ipsilateral breast, and metastasis from an extramammary site.

**Conclusion:**

Although CAM in patients with breast cancer is an uncommon condition, it is still possible to occur. There is a controversy regarding the appropriate management.

## Highlights

• Lymph node metastasis is the most prominent prognostic factor in breast cancer.
• Ipsilateral axillary lymphnode metastasis is the most common site of involvement.
• Contralateral axillary lymphnode metastasis is an unusual finding.
• In this paper, a case of synchronous contralateral axillary lymph node metastasis has been discussed.

## 1 Introduction

Lymph node metastasis is the most prominent prognostic factor in breast cancer, the most prevalent region involved is the ipsilateral axilla, although extra axillary lymph nodes (include the internal mammary chain (IMC), the infraclavicular region, the supraclavicular fossa, and the interpectoral (Rotter's) space) spread can occur [1,2]. CAM is an infrequent clinical finding with a reported incidence of as less as 1.9% [3]. CAM could be encountered at the time of breast cancer which is known as a synchronous CAM, if metastasis occurred after treatment of primary breast cancer, it is regarded as a metachronous CAM [4]. Although till now, it's controversial in the literature whether CAM should be considered as a distant disease or loco-regional metastasis, it has significant effect on the overall survival [5].

The aim of this paper is to report and discuss a case of CAM which seems metachronous initially, but histopathologicaly confirmed as synchronous CAM. The report has been arranged in line with SCARE 2020 guidelines with a brief literature review [6].

## 2 Case report

### 2.1 Patient information

A-44-year old housewife, multiparous, married female was a known case of left breast cancer and treated by Wide Local Excision (WLEx) and Axillary Lymph Node Dissection (ALND I,II,III) five years prior to this presentation (T2,N2,M0, grade III, Triple negative, multifocal invasive ductal carcinoma). At that time, the patient received 8 cycles of chemotherapy and 19 cycles of radiotherapy. She was on regular follow up.

### 2.2 Clinical findings

There was no significant finding. Vital signs were within the normal ranges.

### 2.3 Diagnostic assessment

Hematological tests were normal. Multiple contralateral axillary suspicious lymph nodes were discovered by ultrasound and mammography without any suspicious lesion in both breasts even by MRI scan. Fine Needle Aspiration Cytology (FNAC) from the lesion revealed grade III, Triple negative, invasive ductal carcinoma consistent with metastasis from the left invasive ductal carcinoma.

### 2.4 Therapeutic intervention

The patient was counseled in details and all therapeutic options were discussed. The patient chose and insisted to do bilateral mastectomy and right ALND. The final result of histopathological examination and immunohistochemistry were left breast recurrent 0.5 cm grade III, Triple negative invasive ductal carcinoma, right axillary 3 out of 27 lymph nodes were positive for macro-metastasis without extracapsular extension and no significant pathology in right breast or it's nipple-areolar complex.

### 2.5 Follow up

The post-operative course was uneventful. The patient remained in hospital for one day and discharged home on oral analgesic and antibiotics.

## 3 Discussion

Expectedly breast cancer spreads to the ipsilateral axillary groups of lymph nodes from the primary site. If a cancer is found in the contralateral axilla, three main potential sources should be considered: contralateral spread from the original breast tumor, metastasis from an occult primary in the ipsilateral breast, and metastasis from an extramammary site [7].

CAM is scarce especially when found alone [8]. Both metachronous and synchronous have been reported. The incidence of CAM has been subjected to report biases. It could be overestimated due to the lack of MRI for the diagnosis of occult contralateral tumors in some regions of the world, or the rates may be underestimated because of the lack of regular physical examination and proper investigations [7]. Synchronous CAM is relatively less common than metachronous CAM. In a review by Pasta et al. among 36 reported cases, 22 were metachronous and 14 cases were synchronous [9]. Of the seven cases reported by Huston et al. [10], only one of them was synchronous. The route of spread is unknown, but it's thought to be through the deep lymphatic fascial plexi, although it may spread across the midline through dermal lymphatics [11]. As reported by Kinoshita et al., altered lymphatic spread from the tumor to the contralateral axilla is another route of spread.

There is a conflict in the literature whether CAM should be considered as a distant disease so treated as a stage IV breast cancer rather than a locoregional extension of contralateral breast cancer [5]. There is an aggressive histopathological feature in breast cancer patients with CAM, such as higher tumor grade, lymphovascular invasion, larger primary tumors, ER-negativity, and HER2 overexpression [1]. In this report, the primary breast tumor was a grade III, Triple-negative, multifocal IDC. The median age of patients with CAM has been reported to be between 48 and 50 years [4]. The age of the patient in this study was 44 years.

As reported in the literature, the histopathological and immunohistochemical features of CAM are generally similar to those of primary breast cancer [10]. This finding was confirmed in the current paper. However, as reported by Son et all [12], immunohistochemical analysis of the primary breast lesion revealed ER+, PR+, and HER2– tissue, while the contralateral axillary lymphadenopathy was ER–, PR–, and HER2+. There is a wide variation in histopathological findings with CAM, there may be different disease processes and variability in primary tumor aggressiveness in synchronous and metachronous CAM [7]. Patients with atypical axillary lymph node metastasis should undergo radiological investigations like MRI scan of the breast, it is considered to be a superior investigation in comparison to the mammogram with a reported sensitivity of 85–100% and specificity of 35–95% [13]. In this report, MRI of the contralateral breast was normal.

Management of patients with CAM still controversial. Resection, chemotherapy, and hormonal therapy are among the options to be considered [1]. Palliative treatment should be the priority instead of surgical approach [3]. If there is evidence of systemic disease, then the management should be systemic. Surgery can be performed only in some cases for local control and palliation [1,13] If CAM is the only site of metastasis, therapeutic options include contralateral axillary dissection, which results in excellent axillary control. Contralateral mastectomy is probably not indicated. Because proper workup, including breast MRI, will diagnose most cases with a contralateral primary tumor [1]. In the current study, the MRI of breast was normal, the histopathological examination and immunohistochemistry revealed left breast recurrence (0.5 cm grade III, Triple negative invasive ductal carcinoma). Wang et al. [4] reported that radiotherapy, systemic chemotherapy, and hormone therapy are superior to ALND or mastectomy. However, in a study, most of the patients (89.6%) underwent surgery, about 77.8% administered systemic chemotherapy, the clinician treated the disease as regional rather than distant that's why treated as curative rather than palliative [14].

The long-term survival rate in patients who develop CAM is variable. Wang et al. reported an extremely poor prognosis with a relapsing rate of greater than 70% and a mortality rate of 25% within 2 to 3 years [4]. However, a review by Moossdorff et al. revealed that the survival rate in patients with isolated CAM is 76.6%, and in patient with CAM and ipsilateral breast tumor recurrence is 83.4%, which has a similar prognosis to a regional extension [14].

In conclusion, although CAM in patients with breast cancer is an uncommon condition, it is still possible to occur. There is a controversy regarding the appropriate management.

## Consent

Written informed consent was obtained from the patient for publication of this case report and accompanying images. A copy of the written consent is available for review by the Editor-in-Chief of this journal on request.

## Sources of funding

None is found.

## Provenance and peer review

Not commissioned, externally peer-reviewed.

## Declaration of competing interest

None to be declare.
